# Grazing improves C and N cycling in the Northern Great Plains: a meta-analysis

**DOI:** 10.1038/srep33190

**Published:** 2016-09-12

**Authors:** Xiaoyu Wang, Brian G. McConkey, A. J. VandenBygaart, Jianling Fan, Alan Iwaasa, Mike Schellenberg

**Affiliations:** 1 Ottawa Research and Development Centre, Agriculture and Agri-Food Canada, Ottawa K1A0C6, Canada; 2 Swift-Current Research and Development Centre, A griculture and Agri-Food Canada, Swift Current S9H3X2, Canada

## Abstract

Grazing potentially alters grassland ecosystem carbon (C) and nitrogen (N) storage and cycles, however, the overall direction and magnitude of such alterations are poorly understood on the Northern Great Plains (NGP). By synthesizing data from multiple studies on grazed NGP ecosystems, we quantified the response of 30 variables to C and N pools and fluxes to grazing using a comprehensive meta-analysis method. Results showed that grazing enhanced soil C (5.2 ± 4.6% relative) and N (11.3 ± 9.1%) pools in the top layer, stimulated litter decomposition (26.8 ± 18.4%) and soil N mineralization (22.3 ± 18.4%) and enhanced soil NH_4_^+^ (51.5 ± 42.9%) and NO_3_^−^ (47.5 ± 20.7%) concentrations. Our results indicate that the NGP grasslands have sequestered C and N in the past 70 to 80 years, recovering C and N lost during a period of widespread grassland deterioration that occurred in the first half of the 20^th^ century. Sustainable grazing management employed after this deterioration has acted as a critical factor for C and N amelioration of degraded NGP grasslands and about 5.84 Mg C ha^−1^ CO_2_-equivalent of anthropogenic CO_2_ emissions has been offset by these grassland soils.

Grasslands represent the largest land resource in the world, covering 40% of the earth’s land surface and accounting for 20% of terrestrial production^1^. On a global scale, grasslands store more than 10% of the terrestrial biomass carbon and 10–30% of the global soil organic carbon^1^,^2^. The carbon (C) sequestration rate in grassland soils has been estimated at 0.5 Pg C yr^−1^ globally^3^. Carbon and nitrogen (N) are key factors determining the functions of ecosystems, such as productivity, soil quality and biological cycles^4^. Better understanding the relationships between grassland management and the status of C and N is crucial for the sustainable use of grassland resources and critical in applying the proper management.

Grazing by large mammals is one of the major human activities in uncultivated grasslands around the world^5^. Grazing modifies the C and N cycles that may change the storage of C and N and their processes^6^. Numerous studies have been conducted over the past 50 to 70 years globally, but much uncertainty still exists regarding the effects of grazing on pools and processes of C and N cycling. Mixed results of grazing effects on soil C pools have been found by previous researchers^6^, with studies showing positive^7^, negative^8^, or neutral effects of grazing^9^. The soil N pool is affected by grazing in complex ways, which has increased^10^, decreased^11^ or been maintained by grazing^12^. Grazing affects aboveground net primary production (ANPP) and it has optimized^13^, neutralized^14^ and suppressed^15^ ANPP on North America grasslands. Besides these pool variations, grazing also influences the processes, flux rates and availability of nutrients via feedbacks between plant responses to grazing and nutrient cycling^16^. Dung and urine deposition by grazing animals has influenced the soil N mineralization and immobilization^17^, facilitating rapid substrate decomposition^18^, and increasing the N cycling rate^19^. Concentrations of NH_4_^+^ and NO_3_^−^ in soil usually have increased with grazing in grasslands^20^.

The Northern Great Plains (NGP) is a distinctive region of central North America covering an area of about 1,940,000 km^2^ and having a semiarid to sub-humid continental climate with long, cold winters and short, warm summers^21^,^22^. The NGP experienced a huge disturbance attributed to European settlement beginning in the 1880s, including excessive natural land cultivation and livestock introduction^23^. Between the 1910s and 1930s, the NGP experienced widespread grassland deterioration as the result of poor management and frequent droughts, culminating in the Dust Bowl with severe wind erosion. The C and N was estimated to be at a historical low during this period^23^.

The NGP has been experiencing a restoration under more prudent management practices since the Dust Bowl ended^23^. Currently, about 50% of the NGP remains natural grassland and nearly 100% are grazed by large mammals, principally cattle^24^. These grazing lands are unfertilized, unirrigated, and never reseeded so grazing management is the primary anthropogenic effect controlling the amount and cycling of soil C and N^25^. Considerable experimental research on the effect of grazing management on NGP natural grasslands has been conducted in the past 50–60 years, but uncertainty still exists regarding the overall magnitude and direction of the effects of grazing on various C and N pools and cycles during the restoration. A quantity of synthesized analyses regarding the effect of grazing on grassland ecosystems have been conducted at both global and regional scales on parameters of ANPP^26^ and soil C^6^,^23^,^27^, but it has been noted that the consensus of the grazing effect on grasslands ecosystem C and N pools and cycles for the NGP, particularly during the restoration, is still lacking.

Therefore, in this study, we conducted a meta-analysis of 46 published studies with 864 comparisons on the responses of 30 variables related to various C and N pools, fluxes, descriptive parameters and environmental variables to grazing in the NGP ecosystem. A response ratio (the ratio of a variable in the grazed ecosystem to a native-control) is used here as an index to evaluate the direction and magnitude of the grazing effects^28^,^29^. Overall, the objectives of this study were to evaluate the C and N cycling trends of NGP natural grassland ecosystems during the last 70 to 80 years since the Dust Bowl ended and to quantify the changes in C and N cycles induced by grazing.

## Results

### Carbon and nitrogen pools

**Figure 1:**
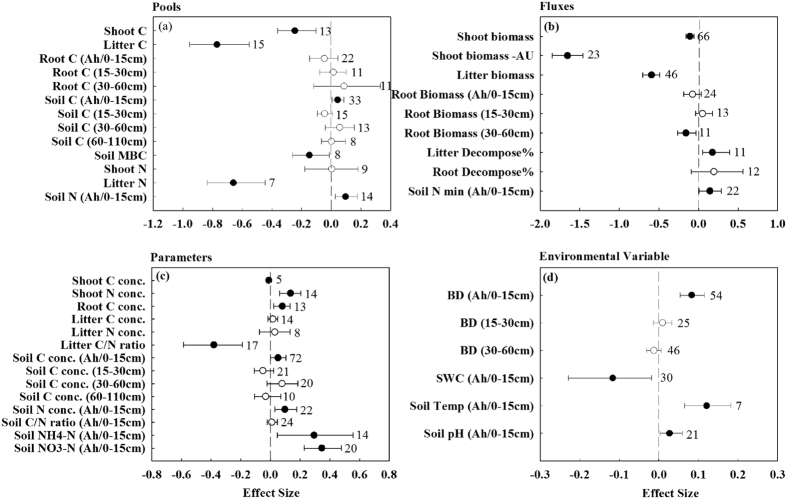
Responses ratio (ln*RR*) of 30 variables related carbon and nitrogen cycles in response to grazing in the NGP natural grazing grassland ecosystem. Bars represent the range of 95% confidence intervals. The vertical dashed line was drawn at *RR* = 0. Response ratios of different depth was reported for pool variables of soil carbon and root carbon, fluxes variables of root biomass, parameter variables of soil carbon concentration and environmental variables of bulk density. MBC, microbial biomass carbon; Shoot biomass ‒ AU, annual utilization of above-ground net primary production by grazers. Soil N min, soil nitrogen mineralization; BD, bulk density. Solid points are significantly response to grazing, and the hollow points are insignificantly response to grazing.

Overall, the percentage changes of 30 variables related to C and N cycles and environmental indicators showed mixed responses to grazing (Fig. 1). Twenty-three out of 30 variables showed significant non-zero responses compared with the control group (*P* < 0.05, Fig. 1). The frequency distributions of most variables followed a normal distribution of Gaussian function (Figs S1–S4), and the *μ*-values from the model of the Gaussian function were consistent with the responding response ratios (Figs S1–S4 vs Fig. 1).

The C stock of the aboveground plant parts was significantly decreased by an average of 19.1 ± 11.4% (mean ± 95% CI, the same below) by grazing while the N stock of the shoots remained unchanged (Fig. 1a). Grazing enhanced soil C and N pools by 5.2 ± 4.6% and 11.3 ± 9.1%, respectively, and such effect was detected in the top 15 cm or the A-horizon depth but not in deeper increments of 15‒30, 30‒60 and 60‒110 cm (Fig. 1a). Carbon and nitrogen pools in litters and the carbon pool in soil microbial biomass (SMBC) presented a significantly negative response to grazing in the NGP (49.7 ± 11.7, 46.6 ± 11.3 and 12.0 ± 11.9%, respectively) (Fig. 1a). The carbon pool in roots showed an insignificant response to grazing across all of the case studies for the increments of Ah/0–15, 15–30 and 30–60 cm (Fig. 1a).

The absolute C changes were +1592.2 ± 1368.7, −186.3 ± 117.5, −592.1 ± 293.6 and −81.6 ± 67.3 kg C ha^−1^ for the pools in soil, shoot, litters and soil microbial biomass and carbon change rate was +71.9 ± 30.9, −8.6 ± 4.5, −28.1 ± 12.3 and −3.2 ± 2.9 kg C ha^−1^ yr^−1^ for the corresponding pools, respectively (Table 1). The absolute change of N stock was −13.6 ± 5.1 and +152.9 ± 151.8 kg N ha^−1^ for litter and soils and their N change rate estimated at −0.9 ± 0.6 and +3.4 ± 3.3 kg N ha^−1^ yr^−1^, respectively (Table 1).

### Fluxes and parameters

Grazing significantly decreased shoot biomass by 8.2 ± 4.6% on the NGP (Fig. 1b). About 78.7 ± 4.4% of the shoot biomass was utilized by cattle each year under heavy grazing (Fig. 1b). Forty-one percent (40.8 ± 6.3%) of litter biomass was removed by grazing while litter decomposition and soil mineralization rates were 26.8 ± 18.4 and 22.3 ± 18.4% higher in the grazed area than in the native control, respectively (Fig. 1b). Root biomass changes were only detected in the 30‒60 cm depth and grazing decreased root biomass by 13.0 ± 11.3% in this layer (Fig. 1b). Among the parameters, shoot N concentration, root C concentration, soil C concentrations (Ah/0‒15 cm depth), soil N concentration (Ah/0‒15 cm depth) and soil NH_4_^+^ and NO_3_^−^ concentrations (Ah/0‒15 cm depth) were 15.4 ± 8.3, 8.9 ± 6.2, 7.6 ± 5.5, 7.1 ± 6.8, 51.5 ± 42.9 and 47.5 ± 20.7% higher in grazed areas compared to native control, while litter C: N and shoot C concentration was reduced by 25.9 ± 14.0% and 0.7 ± 0.4% by grazing (Fig. 1c).

### Environmental variables

The overall effects of grazing on soil bulk density (BD), soil temperature and soil pH were found to be enhanced (Fig. 1d). Compared with the ungrazed control groups, the BD (Ah/0‒15 cm depth), soil temperature and soil pH was significantly increased by an average of 9.3 ± 3.5, 12.8 ± 7.3 and 2.9 ± 1.6% under grazing across NGP grassland ecosystem, respectively (Fig. 1d). Changes in BD was only detected in the top 15 cm or the A-horizon depth but had no effect in other increments of 15‒30 and 30‒60 cm (Fig. 1d). Soil moisture in top soils was significantly reduced by grazing and the changes estimated at 7.1 ± 4.9% for the depth of Ah/0‒15 cm (Fig. 1d).

### Factors influencing C and N cycle responses to grazing

Effect size of grazing on soil C concentration had a significantly negative correlation with mean annual temperature (MAT) on the NGP grasslands (*P* = 0.001, Table 2). Mean annual precipitation (MAP) had a significantly negative correlation with the effect size of grazing on litter biomass, root biomass and root C stock, but had a significantly positive correlation with effect size of grazing on shoot biomass (*P* = 0.03, *P* = 0.001, *P* = 0.041 and *P* = 0.015, respectively, Table 2). Effect size of shoot biomass and soil C stock had a significantly positive correlation with grazing duration but the effect size of soil C concentration in the 15‒30 cm depth had a significantly negative correlation with grazing duration (*P* = 0.046, *P* = 0.034, and *P* = 0.019, respectively, Table 2).

## Discussion

Our analysis demonstrates that the grazing regimes employed after the Dust Bowl has enhanced the C storage in the Northern Great Plain (NGP) in the past 70 to 80 years (Table 2). Large amounts of C have been restored in grassland ecosystems via soils while other C pools of shoot, litters and soil microbes were reduced by grazing (Fig. 1a, Table 1). Although mixed results have been reported for grazing effects on grassland ANPP^14^,^15^,^16^, our results suggest grazing functioned as an overall reduction effect on shoot biomass in the NGP. The decreased C stock in shoots could be attributed to the lowered shoot biomass as well as the lowered shoot C concentrations induced by grazing (Fig. 1b,c). Reduction of litter C stocks was due to both the elimination of the standing dead biomass by grazers and the faster litter decomposition induced by enhanced physical breakdown by animal trampling, and this was supported by many studies conducted on the North American grasslands^30^. Reduction of SMBC induced by grazing might be associated with lower amounts of aboveground plant and litter biomass reserved at the grazed sites, as plant material may have provided available C for microbial communities via root exudates and litter decomposition^31^. In addition, the increased soil density could also create unfavourable physical conditions for microbes that can lead to a reduction in the aerobic microbial activity^32^. The increased soil carbon in the NGP grasslands was detected in the top Ah or 0–15 cm depth but not for the depths below of 15‒30, 30‒60 and 60‒110 cm. This is likely a consequence of this layer being the most biologically active zone and that it receives the largest input of nutrients and organic compounds^27^.

The significantly higher soil C in the grazed grasslands compared to ungrazed suggests that the improved grazing management employed after the Dust Bowl has greatly accelerated C accumulation during the restoration of the NGP. As an example in Alberta, Canada, stocking rate was lowered greatly from 20 ha AU^−1^ in 1955 to 36–40 ha AU^−1^ at present, which allowed the grasslands to recover back to a healthy condition after their severe deterioration^23^. Soils are the main repository of C sequestered and about 1.6 ± 1.4 Mg ha^−1^ (5.8 Mg C ha^−1^ CO_2_-equivalent) C has been stored in the soils of NGP grasslands (Table 1). Carbon sequestration rate was estimated to be 0.07 ± 0.03 Mg C ha^−1^ yr^−1^ which agrees with the range of 0.07–0.30 Mg ha^−1^ yr^−1^ reported for North American grasslands^33^,^34^,^35^. Assuming the remained area of the NGP natural grasslands for grazing is estimated at 97,000,000 ha^21^,^24^, we estimate that, on average, about 6.79 Tg C has been sequestered in the grassland soils of NGP each year during the restoration.

Plant N pool was unchanged under grazing in the study although significant amounts of aboveground shoot biomass was removed by grazers. This was probably due to the significantly enhanced herbage N concentrations in grazed area compared to that in the ungrazed native control (Fig. 1c). The higher plant N concentrations under grazing was attributed to the enhanced plant N uptake as soil nutrient availability increased through manure input, N deposition, plant community composition shifts and the release of the readily available nutrient form which accelerated N mineralization^13^. Nitrogen pools in top soils were enhanced by grazing but they were decreased in the litter (Fig. 1a). Lowered litter N pools was due to the large amounts of litter biomass removed by grazers and the faster litter decomposition^30^. The increased soil N pools was possibly attributed the closely linkage between C and N cycles in terrestrial ecosystems^6^. Increase in C input could promote a significant increase in ecosystem N stock^29^. The N stock in natural grassland ecosystems has been built up over centuries to millennia before grazing utilization occurred. The increases in N stock suggests that grazing can rapidly alter the long-term dynamics of grassland N cycles.

Regarding the fluxes variables, large amounts of litter was removed by grazers in the NGP and root biomass was reduced by grazing in the 30–60 cm depth (Fig. 1b). The decreased belowground biomass suggests that defoliation of plants through long-term season-long grazing reduced energy allocation to the roots, hence reducing root growth and altering root depth distribution^15^. Increased aboveground decomposition rate in our analysis agreed with values reported in many studies, such as Giese *et al*.^36^ who found that grazing increased the shoot decomposition rate by 34% in semi-arid grassland in Inner Mongolia of China. It is well known that decomposition rate processes are governed by environment, substrate quality and activities of decomposers^37^. Grazing significantly increased surface soil temperature and litter quality (expressed as litter C: N ratio) by 13.2 and 25.9%, respectively (Fig. 1d), which might greatly accelerate litter decomposition. The increases in soil net N mineralization were probably also due to the eco-physiological trait differences between native and grazed species^38^. Higher plant and litter N concentrations and lower litter C: N ratio than the native species could lead to higher N mineralization^39^. Such results are supported by both model^16^ and empirical predictions^40^. Enhanced aboveground decomposition rate and N mineralization in the grazed grassland ecosystems suggests that grazing can accelerate nutrient cycling processes in the NGP. The N-enriched plant and litter, rapid decomposition and the higher soil N mineralization could also increase soil NH_4_^+^ and NO_3_^−^ concentrations which can increase N availability^41^. These patterns were correspondingly reflected by the increases in soil NH_4_^+^ and NO_3_^−^ concentrations in the NGP grassland ecosystems in this study (Fig. 1c).

Grazing impacts soil moisture in a complex way which depends on changes of partitioning of available energy received in the canopy surface of a grassland^42^. It could lead to a lowered soil water content (SWC) due to the reduced infiltration by compaction or lead to a higher SWC due to decreases of soil evaporation and plant transpiration^42^. Our results shows livestock grazing on NGP grasslands are functioning as a reduction in SWC in the top soils overall. Reduced soil moisture under grazing is generally attributed to reduced infiltration rates as trampling compacts and seals the soil surface layer^43^. Such impacts can be reflected in increased bulk density of the top layer reported in this study. Grazer trampling and SWC are considered as two important factors as they are directly or indirectly related to the soil microbial activity. Heavy grazing destroys the soil environment and then disturbs the growth and metabolization of microorganisms resulting in lower MBC concentration^44^. Decreases in soil moisture also limited soil microbial activity contributing to lower MBC contents^45^. Such patterns were correspondingly reflected in reduced soil MBC in this study.

Relationships between those variables (only paired-observations >20 were tested) and MAT was only detected for soil C concentration at Ah/0–15 cm depth (*P* < 0.05). Significantly negative relationship between soil C concentration and MAT agree with Burke *et al*.^46^ and suggests that global warming may have the effect of reducing soil organic carbon by stimulating decomposition rates more than net primary production in the surface layer on grazed NGP grassland ecosystem^47^. A strong positive relationship between MAP and shoot biomass is supported by abundant data since water availability acts as the primary constraint to plant productivity in many terrestrial biomes^48^. The decreasing trend of root biomass and root C stock in surface layer as MAP increases indicates that more plant material may transfer from belowground to aboveground in areas with higher rainfall on the grazed NGP. Such trends support existing hypotheses and experimental evidence that root:shoot ratios become lower as moisture availability increases in grassland ecosystems^49^. Positive relationship between shoot biomass and graze duration suggests that the plant productivity in grazed area may reach to an equilibrium or even greater than that in ungrazed area after long term grazing, which supports the hypothesis that grazing can increase aboveground net primary production in a long history of revolution^26^. Soil C stock in the top layer exhibited the same positive relations as grazing duration which is likely due to more shoot biomass accumulated as this carbon is eventually transferred to the soil^47^. Negative effects of graze duration on soil C concentration when sampled to depths of 15‒30 cm agree with McSherry & Ritchie^25^. Soil C sampling to intermediate depths (15‒40 cm) is more sensitive to declines in root biomass in the layer with long term grazing. Although declines in root biomass only detected in depth of 30‒60 cm in current study, root biomass in 15‒30 cm may predicted declines as graze prolonged. The negative relationships between root biomass in 15‒30 cm and graze duration in this study can support this (P < 0.05, this data not reported in Table 2 because paired observations employed were less than 20).

**Figure 2:**
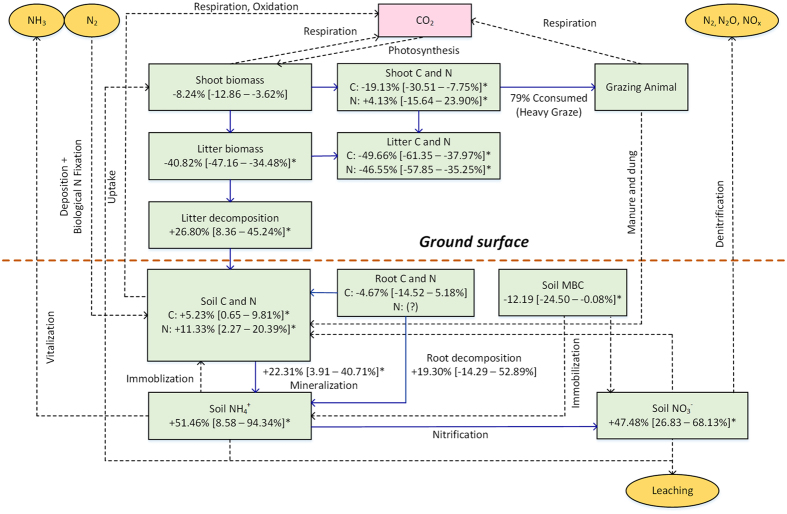
Responses of C and N cycles to grazing in the NGP natural grazing grassland ecosystem. Solid lines represent the carbon and nitrogen processes of corresponding variables synthesized in our meta-analysis. Numbers indicate the mean changes in the C and N cycles with 95% CI. Asterisks indicate statistical significance (*P* < 0.05). MBC: microbial biomass carbon?, not clear for root N pool response to grazing. Dash lines represent the processes not clear for grazing due to not enough data points summarized.

The results from this meta-analysis clearly showed that there was a significant overall direction and magnitude of the response of C and N cycles to graze management on the NGP (Fig. 2). Grazing enhanced not only C and N pools, but also plant decomposition rate and soil N mineralization. These changes indicate that the grazed NGP has been as a C and N sequestration during the restoration process, and there were positive feedbacks between grazing and C and N cycles in the NGP grazed grassland ecosystems since the Dust Bowl ended (Fig. 2). Dashed lines displayed in Fig. 2 represent the processes related to the C and N cycling for which data were absent in the meta-analysis. Absence of C data related to photosynthesis and respiration, and N data related to the processes of deposition, volatilization, immobilization, nitrification and denitrification may not exhibit a complete picture of C and N cycling in grazed NGP ecosystems but could be a valuable topic for future investigation. Overall, all of the changes displayed in the study suggest that grazing profoundly altered NGP ecosystem functioning and processes. Improved grazing management on the NGP has positively contributed to nutrient restoration and acted as a crucial factor for C and N amelioration of degraded North American grasslands.

As in most similar studies, uncertainties exist in this study due to the inherent limitations of the meta-methodology and of experimental manipulations. Firstly, compiled data could have exhibited bias because studies with strong grazing impacts might have been more frequently reported by researchers^28^. Such bias is however difficult to evaluate because of the lack of sufficient data^50^. Secondly, some sub-ecoregions, for example, the Aspen Parkland and Cypress Upland have not attracted such attention from ecologists. The lack of data for these specific sub-ecoregions may bring a small bias to the evaluation of integrated responses of C and N processes to grazing scaled up to the entire NGP. Furthermore, extrapolating the results from independent studies, based on small-scale plot manipulations to an NGP ecosystem scale results in uncertainties. Thirdly, experimental designs, sampling depths, and measurement processes might also have been different from one study to another. For instance, calculations for C and N stocks based on equivalent mass of soils was only done in one study, not adjusting for equivalent mass with depth in the employed studies could also result in a bias in the estimation of changes in C and N stocks when the entire topsoil is not sampled^51^. However, as the first meta-analysis of C and N cycles on the NGP, our findings provide a comprehensive and quantitative understanding of the potential role grazing can have in C and N sequestration for grassland ecosystems of North America.

## Methods

### Data compilation

Publications (see Supporting Information Appendix, Text S1) that studied C and N affected by grazing on the NGP were collected by searching databases of Scopus, Google Scholar, Agricola and Agriculture Index and Web of Science [v.5.14]. Extensive keyword searches were performed by using mixed terms of “grazing”, “ANPP”, “productivity”, “green standing”, “vegetation”, “litter”, “root”, “belowground materials/biomass”, “soil carbon”, “carbon”, “soil nitrogen”, “nitrogen”, “Northern Great Plain”, “Northern prairie”, “Canadian grasslands/prairie” and “fescue grasslands/mixed prairies”. To guarantee we were not missing any publications, we examined the references lists of cited papers within those found by the searches.

To avoid publication bias, a number of criteria were established for a study to be included in this meta-analysis. (1) Treatments of grazed vs. ungrazed had to be conducted in the field sites that were located within the defined Northern Great Plains area and at least one of our considered variables needed to be reported. (2) Experiments had to have been performed on natural grasslands. Studies conducted on pastures with additional management beyond grazing management such as seeding, irrigation or fertilization were excluded. (3) Experiment had grazed by domestic animals such as cattle or sheep were selected. Experimental had grazed by wild ungulates or hogs were excluded. (4) Sites information like vegetation, soils, graze intensity and sampling increments, mean annual precipitation (MAP), mean annual temperature (MAT) and graze duration had to have been recorded and the measurements of variables in ungrazed and grazed treatments were required to be performed at the same temporal and special scales. In order to ensure the data were independent, we made great efforts to exclude duplicate results in different publications. In case with multiple data set published at the same site during different graze duration, the report with the longest record was chosen. In each study, data sampled at different sites, graze intensity, plant community or soils were treated as dependent. Data sampled at one site but in different month were treated as independent then averaged to one paired observation. To avoid bias induced by sampling season, data sampled during the grazed season were selected, usually from May to October each year as most study reported in the North America grasslands. Data sampled prior grazing or in winter time were excluded. Overall, MAT varied from 1.9–8 °C across all selected sites, MAP and graze duration varied between 245–550 cm and 1–85 years, respectively. As a result, 46 publications met the requirements of the criteria.

Data for 30 variables associated with C and N cycles in response to grazing were summarized in the compiled database. Pool variables of C stock in shoot, root, litter, soil and soil microbes and pool variables of N in shoot, litter and soil were tested in the study. Flux variables include shoot biomass (measured in protected cages and in annual utilization), litter biomass, root biomass, litter decomposition rate, root decomposition rate and soil N mineralization. Descriptive variables include shoot C and N concentrations, root C concentration, litter C and N concentrations, litter C:N ratios, soil C and N concentrations, soil C:N ratios in soils, and soil NH_4_^+^ and NO_3_^−^ concentrations. Environmental variables include soil bulk density, soil moisture, soil temperature and soil pH. Other variables such as root N concentration, root N stock, N change induced by plant-associated N fixation and atmospheric N deposition, soil N nitrification, soil N denitrification and soil respiration did not yield sufficient data points for a meta-analysis, therefore they were excluded from the study.

Raw data were either obtained from tables or extracted by digitizing graphs using WebPlotDigitizer (Version 3.8 for Desktop). Carbon and N stocks in shoots, roots and litter were calculated by biomass multiplied by their C and N concentrations if no stock values were directly reported. Carbon and N stocks in soils were only subtracted from those studies that compared grazed plots to ungrazed plots and reported grazing effects on soil carbon and nitrogen density (mass per unit area), or % C together with bulk density, which allowed us to calculate C and N density. Unfortunately, many studies reported only % C or C concentrations in g kg^−1^ or mg g^−1^. These studies were not used in calculating the carbon density with any assigned bulk density (BD) because the effect of grazing on soil BD can cancel or even reverse effects on C and N concentrations, and therefore, may not accurately predict grazing effects on carbon and nitrogen density. Ideally, soil C and N stock in grazed treatments and controls should be compared on an equivalent mass rather than a fixed depth basis because the changes in BD induces by animal traffic may result in bias to stock values^51^. In cases where there were not soil C content but soil organic matter was measured, a correcting factor of 0.58 was used to convert organic matter into soil C content^52^. In our study, soil depth was not adjusted to account for changes unless the authors of the study had already done so. The A-horizon or 0‒15 cm depth usually reported for the top layer in selected studies and other increments were reported as 15‒30, 30‒60, and 60‒110 cm depths, therefore, we established sample increments for the Ah/0–15 cm, 15–30, 30–60, and 60–110 cm depth for soil C. Soil N was only established for Ah/0–15 cm due to lacking data for other increments. Root biomass were established for Ah/0–15 cm, 15–30 and 30–60 cm depths. Data reported in different sub-depths within above defined increments were interpolated to the corresponding depth by summing the C and N stocks or root data in sub-depths together. In total, the constructed database consisted of 864 lines of entries of paired observations.

### Meta-Analysis

We used natural log of the response ratio (*RR*) defined as “effect size” as a metric for the response of C and N variables to grazing^28^. For a given variable, *RR* was calculated as the ratio of its value in the grazed treatment group (*X*_*G*_) to that in the control un-grazed group (*X*_*UG*_):


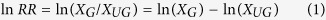


if the *X*_*G*_ and *X*_*UG*_ populations are normally distributed and both are greater than zero, ln (*X*_*G*_/*X*_*UG*_) is assumed approximately normally distributed with a mean equal to the true response ratio^29^. For improved interpretation, the results were reported as the percentage change estimated by (*e*^*lnRR*^ − 1) × 100%. Outliers were exclude by the range of Mean ± 2 × STDEV.

Ideally, the meta-analysis of ln*RR* should be weighted by the sample size or variance for each study. But in our database, most studies did not report the variance in any form. Therefore, to include as many studies as possible we applied un-weighted meta-analysis in which all studies in the dataset were assigned an equal variance^53^. In an un-weighted meta-analysis, mean effect sizes and confidence intervals are generated by bootstrapping, which estimates distributional statistics by iteratively permuting and resampling the data set. If the 95% CI values of the effect size for a variable did not overlap with 0, the effect of grazing on variables was considered to differ significantly between the two treatments. We performed our meta-analyses by using MetaWin 2.1 with 4999 iterations^54^.

To test the normality of the constructed dataset for each variable, the frequency distribution of ln*RR* is assumed to follow a normal distribution and to be fitted by a Gaussian function^29^:


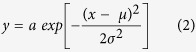


where *x* is the mean of ln*RR* in an individual interval, *y* is the frequency (i.e., number of ln*RR* values) in an interval, *a* is a coefficient showing the expected number of ln*RR* values at *x* = *μ*, *μ* and *σ* are mean and variance of the frequency distributions of ln*RR*, and *e* is the base of exponent. The fitting of Gaussian functions were plotted by using SigmaPlot 12.0. In addition, we also applied regression analyses to examine the relationship between ln*RR* and environmental (MAP and MAT) and graze duration as forcing factors. Variables with >20 observations were selected.

## Additional Information

**How to cite this article**: Wang, X. *et al*. Grazing improves C and N cycling in the Northern Great Plains: a meta-analysis. *Sci. Rep.* **6**, 33190; doi: 10.1038/srep33190 (2016).

## Supplementary Material

Supplementary Information

## Figures and Tables

**Table 1:** Effect of grazing on various C and N stock, C and N change rate and CO_2_ equivalent in the NGP.

Variable	Depth	Stock	95% CI	CO_2__equivalent	Stock Rate	95% CI	CO_2__equivalent
Carbon pools	cm	kg ha^−1^	±	kg ha^−1^	kg ha^−1^ yr^−1^	±	kg ha^−1^ yr^−1^
Shoot		**−186.3**	**117.5**	−683.2	**−8.6**	**4.5**	−31.5
Litter		**−592.1**	**293.6**	−2171.2	**−28.1**	**12.3**	−103.1
Root	Ah/0–15	13.4	426.5	−49.1	5.3	19.4	19.4
Soil	Ah/0–15	**1592.2**	**1368.7**	5838.6	**71.9**	**30.9**	263.7
Soil microbial biomass C	Ah/0–15	**−81.6**	**67.3**	−299.2	**−3.2**	**2.9**	−11.7
Nitrogen pools							
Shoot		0.9	4.0	NA	0.4	0.6	NA
Litter		**−13.6**	**5.1**	NA	**−0.9**	**0.6**	NA
Soil	Ah/0–15	**152.9**	**151.8**	NA	**3.4**	**3.3**	NA

CO_2__equivalent converted by pools carbon mass multiply the atom mass ratio of CO_2_/C (44/12).

**Table 2:** Percentage of change of variables related to carbon and nitrogen cycles in response to grazing on the Northern Great Plains.

Variable	Depth (cm)	Intercept	Slope	n	r^2^	P
MAT						
Shoot biomass		−0.127	0.003	66	0.003	0.648
Litter biomass		−0.509	−0.013	46	0.021	0.338
Root biomass	Ah/0–15	−0.209	0.028	24	0.024	0.470
Root C stock	Ah/0–15	−0.062	0.004	22	0.001	0.895
Soil C stock	Ah/0–15	0.091	−0.009	33	0.007	0.649
Soil C conc.	Ah/0–15	0.299	**−0.052**	72	0.141	**0.001**
	15–30	−0.114	0.013	21	0.140	0.624
	30–60	0.190	−0.023	20	0.014	0.618
Soil N conc.	Ah/0–15	−0.162	0.041	22	0.143	0.083
Soil C: N	Ah/0–15	0.077	−0.013	24	0.033	0.394
Soil N min.	Ah/0–15	0.636	−0.092	22	0.129	0.119
Soil NO3-N	Ah/0–15	0.252	0.018	20	0.001	0.888
MAP						
Shoot biomass		−0.483	**0.001**	66	0.075	**0.015**
Litter biomass		0.050	**−0.002**	46	0.079	**0.030**
Root biomass	Ah/0–15	0.814	**−0.002**	24	0.342	**0.001**
Root C stock	Ah/0–15	0.354	**−0.001**	22	0.203	**0.041**
Soil C stock	Ah/0–15	0.054	0.000	33	0.000	0.913
Soil C conc.	Ah/0–15	0.024	0.000	72	0.001	0.812
	15–30	0.380	−0.001	21	0.140	0.095
	30–60	0.675	−0.002	20	0.168	0.073
Soil N conc.	Ah/0–15	0.094	0.000	22	0.002	0.850
Soil C: N	Ah/0–15	0.023	0.000	24	0.002	0.847
Soil N min.	Ah/0–15	1.120	−0.002	22	0.120	0.114
Soil NO3-N	Ah/0–15	−0.194	0.001	20	0.115	0.144
Duration						
Shoot biomass		−0.158	**0.003**	66	0.061	**0.046**
Litter biomass		−0.533	−0.003	46	0.019	0.364
Root biomass	Ah/0–15	−0.027	−0.003	24	0.020	0.513
Root C stock	Ah/0–15	−0.037	0.000	22	0.117	0.128
Soil C stock	Ah/0–15	−0.020	**0.002**	33	0.137	**0.034**
Soil C conc.	Ah/0–15	0.124	−0.002	72	0.047	0.067
	15–30	0.072	**−0.003**	21	0.256	**0.019**
	30–60	0.100	−0.001	20	0.004	0.779
Soil N conc.	Ah/0–15	−0.016	0.002	22	0.070	0.235
Soil C: N	Ah/0–15	0.003	0.000	24	0.001	0.905
Soil N min.	Ah/0–15	0.072	0.003	22	0.066	0.248
Soil NO3-N	Ah/0–15	0.212	0.004	20	0.087	0.207

The regression analysis was based on ln*RR* (Variable X) = *Intercept *+ *Slope* *X, where X is the independent variables, *n* is sample size, *r^2^* is determinant coefficient and *P* is probability of the regression relationship to be statistically significant. Values in bold are statistically significant from zero. Variable with >20 observations were selected.
