# Supplementary material for: Grazing improves C and N cycling in the Northern Great Plains: a meta-analysis
Source: Sci Rep. 2016 Sep 12;6:33190. doi: 10.1038/srep33190 (PMC5018814; doi:10.1038/srep33190)

Supporting Information Appendix

Grazing improves C and N cycling in the Northern Great Plains: a meta-analysis

Xiaoyu Wang[1], Brian. G. McConkey[2,*], A. J. VandenBygaart[1], Jianling Fan[2], Alan Iwaasa[2], Mike Schellenberg[2]

[1]Ottawa Research and Development Centre, Agriculture and Agri-Food Canada, Ottawa K1A0C6, Canada.

[2]Swift-Current Research and Development Centre, Agriculture and Agri-Food Canada, Swift Current S9H3X2, Canada.

*Corresponding author: Brian McConkey. 1 Airport Road, Swift Current, Saskatchewan S9H3X2. Telephone: 306-770-4498. Fax: 306-770-4411. Email: brian.mcconkey@agr.gc.ca

Text S1. A list of 46 papers from which the data were extracted for this meta-analysis.

1. Bai, Y, Abouguendia, Z, & Redmann, RE (2001) Relationship between plant species diversity and grassland condition. *Journal of Range Management*, 54: 177–183.
2. Biondini ME (1996) Grazing frequency and ecosystem processes in a Northern Mixed Prairie, USA. *Ecological Application* 6(1): 239–256.
3. Biondini ME, Patton BD, Nyren PE (1998) Grazing intensity and ecosystem processes in a Northern Mixed Prairie, USA. *Ecological Application* 8(2): 469–479.
4. Brand MD, Goetz H (1986) Vegetation of exclosures in southwestern North Dakota. *Journal of Range Management*. 39(5): 434–437.
5. Bork E, Willms WD, Tannas S, Alexander M (2012) Seasonal patterns of forage availability in the Fescue Grasslands under contrasting grazing histories. *Rangeland Ecology & Management* 65(1): 47–55.
6. Chanasyk, DS, & Naeth, MA (1995) Grazing impacts on bulk density and soil strength in the foothills fescue grasslands of Alberta, Canada. *Canadian Journal of Soil Science* 75(4): 551–557.
7. Dormaar JF, Johnston A, Smoliak S (1977) Seasonal variation in chemical characteristics of soil organic matter of grazed and un-grazed Mixed Prairie and Fescue grasslands. *Journal of Range Management* 30(3): 195–198.
8. Dormaar JF, Johnston A, Smoliak S (1984) Seasonal changes in carbon content, and dehydrogenase, phosphatase, and urease activities in Mixed Prairie and Fescue grassland Ah horizon. *Journal of Range Management* 37(1): 31–35.
9. Dormaar JF, Smoliak S, Willms WD (1989) Vegetation and soil responses to short-duration grazing on fescue grasslands. *Journal of Range Management* 42(3): 252–256.
10. Dormaar JF, Smoliak S, and Willms WD (1990a) Distribution of nitrogen fractions in grazed and ungrazed fescue grassland Ah Horizons. *Journal of Range Management* 43(1): 6–9.

11. Dormaar JF & Willms WD (1990b) Sustainable production from the rough fescue prairie. *Journal of Soil and Water Conservation* 45(1): 137–140.

12. Dormaar JF, Willms WD (1992) Water-extractable organic matter from plant litter and soil of rough fescue grassland. *Journal of Range Management* 45(2): 152–158.

13. Dormaar JF, Adams BW, Willms WD (1994) Effect of grazing and abandoned cultivation on a *Stipa-Boutelous* community. *Journal of Range Management* 47(1): 28–32.

14. Dormaar JF, Adams BW, Willms WD (1997) Impacts of rotational grazing on Mixed Prairie soils and vegetation. *Journal of Range Management* 50(6): 647–651.

15. Dormaar JF & Willms WD (1998) Effect for forty-four years of grazing on fescue grassland soils. *Journal of Range Management* 51(1): 122–126.

16. Frank AB, Tanaka DL, Hofmann L, Follett RF (1995) Soil carbon and nitrogen of Northern Great Plains grasslands as influenced by long-term grazing. *Journal of Range Management* 48(5): 470–474.

17. Haferkamp, MR & MacNeil, MD (2004) Grazing effects on carbon dynamics in the northern mixed-grass prairie. *Environmental Management* 33(1): S462–S474.

18. Haferkamp MR, MacNeil MD, Grings EE (2005) Predicting nitrogen content in the Northern Mixed-Grass prairie. *Rangeland Ecology & Management* 58(2): 155–160.

19. Henderson DC (2000) Carbon storage in grazed prairie grasslands of Alberta. M. Sc. Thesis, Department of Renewable Sources, University of Alberta, Edmonton, Alb.

20. Hewins, DB, et al. (2015). Grazing, regional climate and soil biophysical impacts on microbial enzyme activity in grassland soil of western Canada. *Pedobiologia* 58(5): 201–209.

21. Ingram LJ, et al. (2008) Grazing impacts on soil carbon and microbial communities in a Mixed-Grass ecosystem. *Soil Science Society of America Journal* 72(4): 939–948.

22. Johnston A (1961) Comparison of lightly grazed and ungrazed range in the fescue grassland of southwestern Alberta. *Canadian Journal of Plant Science* 41(3): 615–622.

23. Li CL, et al. (2012) Changes in soil C, N, and P with long-term (58 years) cattle grazing on rough fescue grassland. *Journal of Soil Science and Plant Nutrition* 175: 339–344.

24. Li CL, Hao X, Willms WD, Zhao MG, Han GD (2009) Seasonal response of herbage production and its nutrient and mineral contents to long-term cattle grazing on a Rough Fescue grassland. *Agriculture, Ecosystems & Environment* 132: 32–38.

25. Lodge RW (1954) Effects of grazing on the soils and forage of mixed prairie in southwestern Saskatchewan. *Journal of Range Management* 7:166–170.

26. Lecain DR, Morgan JA, Schuman GE, Reeder JD, Hart RH (2000) Carbon exchange rates in grazed and un-grazed pastures of Wyoming. *Journal of Range Management* 53(2): 199–206.

27. MacNeil MD, Haferkamp MR, Vermeire LT, Muscha JM (2008) Prescribed fire and grazing effects on carbon dynamics in a northern mixed-grass prairie. *Agriculture, Ecosystems & Environment* 127: 66–72.

28. Manley JT, Schuman GE, Reeder JD, Hart RH (1995) Hart Rangeland soil carbon and nitrogen responses to grazing. *Journal of Soil and Water Conservation* 50(3): 294–298.

29. Mousel, EM, Schacht, WH, Reece, PE, Herron, AE & Koehler, A (2011) Vegetation production responses to October grazing in the Nebraska Sandhills. *Rangeland Ecology & Management* 64(2): 208–214.

30. Naeth MA, Bailey AW, Pluth DJ, Chansyk DS, Hardin RT (1991) Grazing impacts on litter and soil organic matter in Mixed Prairie and Fescue grassland ecosystems of Alberta. *Journal of Range Management* 44(1): 7–12.

31. Naeth MA, Pluth DJ, Chanasyk DS, Bailey AW, Fedkenheuer AW (1990) Soil compacting impacts of grazing in mixed prairie and fescue grassland ecosystems of Alberta. *Canadian Journal of soil Science* 70:157–167.

32. Patton, BD, Dong, X, Nyren, PE & Nyren, A (2007) Effects of grazing intensity, precipitation, and temperature on forage production. *Rangeland Ecology & Management* 60(6): 656–665.

33. Reinhart, KO, Nichols, KA, Petersen, M & Vermeire, LT (2015) Soil aggregate stability was an uncertain predictor of ecosystem functioning in a temperate and semiarid grassland. *Ecosphere* 6(11): 1–16.
34. Rogers WM, Kirby DR, Nyren PE, Patton BD, Dekeyser ES (2005) Grazing intensity effects on Northern Plains Mixed-Grass prairie. *The prairie naturalist* 37(2): 73–83.
35. Schuman GE, Reeder JD, Manley JT, Hart RH, Manley WA (1999) Manley impact of grazing management on the carbon and nitrogen balance of a Mixed-Grass rangeland. *Ecological Application* 9(1): 65–71.
36. Shariff AR, Biondini ME, Grygiel CE (1994) Grazing intensity effects on litter decomposition and soil nitrogen mineralization. *Journal of Range Management* 47(6): 444–449.
37. Shrestha G, Stahl PD (2008) Carbon accumulation and storage in semi-arid sagebrush steppe: Effects of long-term grazing exclusion. *Agriculture, Ecosystems & Environment* 125:173–181.
38. Smoliak S (1965) A comparison of ungrazed and lightly grazed *Stipa-Bouteloua* prairie in southeastern Alberta. *Canadian Journal of Plant Science* 45: 270–275.
39. Smoliak S, Dormaar JF, Johnston A (1972) Long-term grazing effects on Stipa-Bouteloua Prairie soils. *Journal of Range Management* 25: 246–250.
40. Stohlgren, TJ, Schell, LD, & Vanden Heuvel, B (1999) How grazing and soil quality affect native and exotic plant diversity in Rocky Mountain grasslands. *Ecological applications* 9(1): 45–64.
41. Welker, JM, Fahnestock, JT, Povirk, KL, Bilbrough, CJ, & Piper, RE (2004) Alpine grassland CO_2 exchange and nitrogen cycling: grazing history effects, Medicine Bow Range, Wyoming, USA. Arctic, antarctic, and alpine research, 36(1): 11–20.
42. Wienhold BJ, Hendrickson JR, Karn JF (2001) Pasture management influences on soil properties in the Northern Great Plains. *Journal of Soil and Water Conservation* 56(1): 27–31.
43. Willms WD, Dormaar JF, Schaalje GB (1988) Stability of grazed patches on rough fescue grasslands. *Journal of Range Management* 41(6):503–508.

44. Willms, WD, Smoliak, S, & Dormaar, JF (1990) Vegetation response to time-controlled grazing on mixed and fescue prairie. *Journal of Range Management* 43(6): 513–517.

45. Willms WD, Dormaar JF, Adams BW, Douwes HE (2002) Response of the Mixed Prairie to Protection from Grazing. *Journal of Range Management* 55(3): 210–216.

46. Yang, X, Guo, X, & Fitzsimmons, M (2012) Assessing light to moderate grazing effects on grassland production using satellite imagery. *International journal of remote sensing* 33(16): 5087–5104.

Table S1. Overview of grazing studies in North Great Plain included in the meta-analysis. Study information includes mean annual precipitation (MAP), mean annual temperature (MAT), start and end year of experiments, plant community, and soil information and graze intensity.

Con.	Reference	Location	Coordinates	MAP	MAT	Initial	End	Dura.	Plant community	Soils	Intensity
CA	Li *et al.* 2009	Stavely, AB	50°12'N, 113°54'W	494	5.6	1949	2007	58	*Rough fescue*	Black Chernozemic	M, H
CA	Li *et al.* 2012	Stavely, AB	50°12'N, 113°54'W	502	5.6	1949	2007	58	*Rough fescue*	Black Chernozemic	M, H
CA	Dormaar *et al.* 1977	Manyberries, AB	49°42'N, 110°70'W	310	4.5	1928	1973	45	*Stipa-Bouteloua*	Brown Solonetzic	H
		Stavely, AB	50°12'N, 113°57'W	500	5.6	1949	1973	24	*Rough fescue*	Black Chernozemic	H
CA	Dormaar *et al.* 1984	Manyberries, AB	49°42'N, 110°70'W	310	4.5	1928	1978-1980	50-52	*Stipa-Bouteloua*	Brown Solonetzic	H
		Stavely, AB	50°12'N, 113°57'W	500	5.6	1949	1978-1980	28-31	*Rough fescue*	Black Chernozemic	H
CA	Dormaar *et al.* 1989	Fort Macleod, AB	49°47'N, 113°39'W	450	5.7	1981	1983-1986	2-5	*Rough fescue*	Black Chernozemic	H
CA	Dormaar *et al.* 1990a	Stavely, AB	50°N, 113°W	500	5.6	1949	1986	37	*Rough fescue*	Black Chernozemic	L, H
CA	Dormaar *et al.* [illegible]	Stavely, AB	50°N, 113°W	550	5.6	1949	1989	40	*Rough fescue*	Black Chernozemic	L, M, H
CA	Dormaar *et al.* 1992	Stavely, AB	50°N, 113°W	500	5.6	1949	1988	39	*Rough fescue*	Black Chernozemic	L, H
CA	Dormaar *et al.* 1994	Lethbridge, AB	49°46'N, 112°46'W	425	5.7	1978	1991	13	*Stipa-Bouteloua*	Dark Brown Chernozemic	M, H
CA	Dormaar *et al.* 1997	Brooks, AB	50°37'N, 112°10'W	335	4	1987	1992	5	*Stipa-Bouteloua-Agropyron*	Brown Solonetzic	H
CA	Dormaar *et al.* 1998	Southwest AB	49°N, 112°W	550	5	1949	1992	43	*Rough fescue*	Black Chernozemic	L, H
CA	Willms *et al.* 1988	Southwest AB	49°N, 112°W	614	5	1949	1985-1987	36-38	*Rough fescue*	Black Chernozemic	H
CA	Willms *et al.* 1990	Taber, AB	49°N, 112°W	382	6.5	1982	1983-1987	1-5	*Stipa-Bouteloua*	Brown chenozemic	M
		Fort Macleod, AB	49°N, 113°W	435	5.7	1982	1983-1987	1-5	*Rough fescue*	Black Chernozemic	M
CA	Willms *et al.* 2002	Onefour, AB	49°07'N, 110°28'W	332	4.6	1927	1993	66	*Stipa-Bouteloua*	Brown Chernozemic [illegible]	M
		Onefour, AB	49°07'N, 110°28'W	332	4.6	1927	1993	66	*Stipa-Bouteloua*	Brown Solonetzic	M
CA	Henderson 2000	StavelyB, AB	50°N, 113°W	476	5.6	1949	1999	50	*Rough fescue*	Black Chernozemic	L
		StavelyC, AB	50°N, 113°W	476	5.6	1949	1999	50	*Rough fescue*	Black Chernozemic	L
		Twinriver, AB	49°N, 112°W	390	5.3	1970	1999	29	*Rough fescue*	Black Chernozemic	M
		Picturebutte, AB	49°N, 112°W	374	4.7	1978	1999	21	*Stipa-Bouteloua*	Dark Brown Chernozemic [illegible]	H
		Turin, AB	49°N, 112°W	360	4.7	1976	1999	23	*Stipa-Bouteloua*	Dark Brown Chernozemic	H
		Hays, AB	50°N, 111°W	328	4.6	1979	1999	20	*Stipa-Bouteloua*	Brown chenozemic	M
		OnefourA, AB	49°N, 110°W	351	4.6	1948	1999	51	*Stipa-Bouteloua*	Brown chenozemic	L
		OnefourB, AB	49°N, 110°W	351	4.6	1927	1999	72	*Stipa-Bouteloua*	Brown chenozemic	L
		OnefourC, AB	49°N, 110°W	351	4.6	1927	1999	72	*Stipa-Bouteloua*	Brown chenozemic	L
CA	Johnston 1961	Stavely, AB	50°N, 113°W	609	5.6	1949	1961	12	*Rough fescue*	Black Chernozemic	L
CA	Smoliak 1965	Manyberries, AB	49°42'N, 110°70'W	310	4.5	1928/1948	1962	13/33	*Stipa-Bouteloua*	Brown Solonetzic	L
CA	Smoliak *et al.* 1972	Manyberries, AB	49°42'N, 110°70'W	310	4.5	1951	1970	19	*Stipa-Bouteloua*	Brown Solonetzic	L, M, H
CA	Neath *et al.* 1990	Brooks, AB	51°N, 112°W	355	4	1964	1985	21	*Stipa-Bouteloua-Agropyron*	Brown Solonetzic	L,H
		Kinshella, AB	53°N, 111°W	422	2	1973	1985	12	*Rough fescue*	Black Chernozemic	L,H
		Stavely, AB	50°N, 114°W	550	5.6	1949	1985	36	*Rough fescue*	Black Chernozemic	L,H
CA	Neath *et al.* 1991	Brooks, AB	51°N, 112°W	355	4	1964	1985	21	*Stipa-Bouteloua-Agropyron*	Brown Solonetzic	L,H
		Kinshella, AB	53°N, 111°W	422	2	1973	1985	12	*Rough fescue*	Black Chernozemic	L,H
		Stavely, AB	50°N, 114°W	550	5.6	1949	1985	36	*Rough fescue*	Black Chernozemic	L,H
CA	Chanasyk *et al.* 1995	Stavely, AB	50°N, 114°W	550	5.6	1949	1998-2000	49-51	*Rough fescue*	Black Chernozemic	H,VH

CA	Lodge 1954	SK	0°N, 109°W	367	4.2	1950	1951	1	*Stipa-Bouteloua*	Dark Brown Chernozemic	L, M, H
CA	Bork *et al.* 2012	Cypress Hills, AB	49°34'N, 110°5'W	245	3.1	-	-	33	*Rough fescue*	Black Chernozemic	L
		Dutch Creek, AB	49°54'N, 114°23'W	370	5.5	-	-	57+	*Rough fescue*	Black Chernozemic	L
		Castle River, AB	49°25'N, 114°19'W	398	4.8	-	-	57	*Rough fescue*	Black Chernozemic	L
		Ross Lake, AB	49°07'N, 112°53'W	257	3.5	-	-	-	*Rough fescue*	Black Chernozemic	L
		Waldron, AB	49°48'N, 114°06'W	370	2.9	-	-	20	*Rough fescue*	Black Chernozemic	L
CA	Hewins et al., 2015	Foothills, AB	.	470	3.9	.	.	40	*Fescues*	.	L-M
		Parkland, AB	.	441	2.3	.	.	40	*Fescues*	.	L-M
		Prairie, AB	.	394	4.4	.	.	40	*mixed-grass*	.	L-M
CA	Yang et al., 2012	GNP, SK	49°12´N,107°24´W	340	3.6	1986	2003	17	*Stipa-Bouteloua-Agropyron*	Dark-chernozemic	L
CA	Baietal2001	GNP, SK	49°10´N,107°30´W	340	3.6	.	.	5	*Agronpyron-Bouteloua-Stipa*	Brown-Chernozemic	M
		Matador, SK	50°30´N,107°30´W	340	3.3	.	.	30	*Agronpyron-Bouteloua-Eurotia*	Brown-Chernozemic	L
		Parkbeg, SK	50°20´N,106°10´W	358	4.1	.	.	7	*Stipa-Agronpyron-Koeleria*	Brown-Chernozemic	H
		Arena, SK	49°20´N,109°05´W	331	3.4	.	.	6	*Agronpyron-Stipa-Artemsia*	Brown-Chernozemic	M
		Chaplin, SK	50°30´N,106°40´W	357	3.6	.	.	15	*Stipa-Stipa-Agronpyron*	Brown-Chernozemic	H
		Kerr, SK	49°55´N,105°40´W	353	3.1	.	.	7	*Agronpyron-Artemsia-Bouteloua*	Brown-Chernozemic	H
		Estevan, SK	49°05´N,103°05´W	400	3.3	.	.	10	*Calamovilfa-Stipa-Carex*	Dark-Brown-Chernozemic	L
		Glenavon, SK	50°00´N,103°00´W	432	1.9	.	.	6	*Fescue-Poa-Stipa*	Brown-Chernozemic	M
		Glenavon, SK	50°00´N,103°00´W	432	1.9	.	.	6	*Fescue-Poa-Stipa*	Brown-Chernozemic	H
US	Manley *et al.* 1995	Cheyenne, WY	41°11'N, 104°53'W	338	8	1982	1993	11	*Pascopyrum-Stipa-Bouteloua*	Aridic Argiustoll	L,H
US	Schuman *et al.* 1999	Cheyenne, WY	41°11'N, 104°53'W	384	8	1982	1993	11	*Pascopyrum-Stipa-Bouteloua*	Aridic Argiustoll	L,H
US	Ingram *et al.* 2008	Cheyenne, WY	41°11'N, 104°53'W	425	8	1982	1993	11	*Pascopyrum-Stipa-Bouteloua*	Aridic Argiustoll	L,H
US				425	8	1982	2003	21	*Pascopyrum-Stipa-Bouteloua*	Aridic Argiustoll	L,H
US	Shrestha *et al.* 2008	Granite Mountain	43°N, 108°W	213	6.9	1962	2004	42	*Agropyron*	Ustic Haplargid	L
US		Upper Government	43°N, 108°W	200	6.9	1958	2004	46	*Agropyron*	Ustic Haplargid	L
US		Shoshoni #8	43°N, 108°W	203	6.9	1964	2004	40	*Agropyron-Stipa*	Ustic Haplargid	L
US		Shoshoni #9	43°N, 108°W	203	6.9	1964	2004	40	*Bouteloua*	Ustic Haplargid	L
US	Frank *et al.* 1995	Mandan, ND	46°46'N, 100°50'W	410	5.8	1916	1991	75	*Stipa-Bouteloua*	Typic Haplorborolls	M,H
US	Wienhold *et al.* 2001	Morton, ND	46°46'N, 100°50'W	410	4	1932	1997	65	*Stipa-Bouteloua*	Typic Haplorborolls	M,H
US	Rogers *et al.* 2005	Streeter, ND	46°N, 101°W	458	5.1	1988	2000	12	*Poa-Bouteloua*	Typic Argiudtoll	M,H
US	Haferkamp *et al.*	Miles city, MT	46°22'N, 105°5'W	343	7.7	1996	1997-1998	1-3	*Bouteloua-Stipa-Agropyron*	Aridic Argiustoll	H
US	Haferkamp *et al.*	Miles city, MT	46°22'N, 105°5'W	343	7.7	1996	1997-1998	1-3	*Bouteloua-Stipa-Agropyron*	Aridic Argiustoll	H
US	MacNeil *et al.* 2008	Miles city, MT	46°22'N, 105°5'W	340	7.7	2004	2005	1-2	*Bouteloua-Stipa-Agropyron*	Aridic Argiustoll	H
US	Biondidi *et al.* 1996	Dann county, ND	Site 1, 47°N, 102°W	400	4.6	1984	1989	5	*Agropyron-Stipa-Bouteloua*	Veban-Parshall	M
			Site 2, 47°N, 102°W	400	4.6	1984	1989	5	*Agropyron-Stipa-Bouteloua*	Cabba-Cohagen-Wayden	M
			Site 3, 47°N, 102°W	400	4.6	1984	1989	5	*Agropyron-Stipa-Bouteloua*	Amor-Morton-Farland-	M
US	Biondidi *et al.* 1998	Streeter, ND	46°N, 99°W	446	5.1	1979	1988-1995	9-16	*Agropyron-Stipa-Bouteloua*	Typic Argiudtoll	M,H
US	Shariff *et al.* 1994	Streeter, ND	46°N, 99°W	432	5.1	1979	1989	10	*Agropyron-Stipa-Bouteloua*	Typic Argiudtoll	M,H
US	Brand & Goetz 1986	Little Missouri, ND	46°N, 100°W	356	5.3	1976	1978	2	*Bouteloua-Stipa-Agropyron*	Typic Argiudtoll	H
US	LeCain *et al.* 2000	Cheyenne, WY	41°11'N, 104°54'W	384	8	1982	1995	13	*Pascopyrum-Stipa-Bouteloua*	Aridic Argiustoll	L,H

US	Stohlgren *et al*. 1999	Bighorn-Basin, WY	.	376	6.7	.	.	14	*Aronpyron-Artemisia*	.	M
		Charles-Russell, MT	.	134	6.9	.	.	30	*Aronpyron-Artemisia*	.	M
		Grand-Teton, WY	.	418	3.2	.	.	33	*Aronpyron-Artemisia*	.	M
US	Welker *et al.* 2004	Libby-Flats, WY	41°20´N,106°17´W	316	5.7	.	.	45	*Deschampsi-Poa*	Fine-loamy	M
		Hay-Creek, WY	41°20´N,106°17´W	359	7.2	.	.	85	*Festuca-Bromus*	Fine-loamy	M
US	Patton *et al*. 2007	CGREC-Overflow, ND	46°46´N,99°28´W	454	2	1989	2005	16	*poa-Bromus*	Sandy	L,M,H
US		CGREC-Silty, ND	46°46´N,99°28´W	454	2	1989	2005	16	*Poa-Carex*	Fine	L,M,H
US	Mousel *et al*. 2011	GSL-Whitman, NB	42°07´N,101°26´W	468	13.3	1997	2002	5	*Bluestem-Needle*	Vslentine fine sands	L,M,H
US			42°07´N,101°26´W	468	13.3	1997	2002	5	Cool-season	Vslentine fine sands	L,M,H
US	Reinhart *et al*. 2015	ARSFKL-RRL, MT	46°18´21"N,105°58´43"W	340	7.7	1999	2013	14	*Hesperostipa-Bouteloua-Carex*	Frigid Aridic Argiustolls	L,M

Table S2. Response ratio (*RR*) and numbers of paired observations (in parentheses) of variables related to C and N pools and fluxes extracted from each paper. Shoot-Bio presents aboveground net primary production sampled at cage protected; Shoot-AU presents aboveground net primary production annual utilization; Deco. means decomposition; Min. means mineralization; conc. means concentration; Temp means temperature; BD means bulk density.

Response ratios for C pool, N pool and Fluxes (to be continued)

No	Reference	C Pool					N pool			Fluxes						
		**Shoot C**	**Litter C**	**Root C**	**Soil**	**Soil MBC**	**Shoot N**	**Litter N**	**Soil N**	**Shoot-Bio**	**Shoot-AU**	**Litter biomass**	**Root biomass**	**Litter Deco.**	**Root Deco.**	**Soil Min.**
1	Li *et al*. 2009									0.3075<br>(2)		-0.5164<br>(1)				
2	Li *et al*. 2012				-0.0105<br>Ah/0-15<br>(2)				-0.0111<br>Ah/0-15<br>(2)							
					-0.1039<br>15-30<br>(2)											
					-0.0419<br>30-60<br>(2)											
3	Dormaar *et al*. 1977															
4	Dormaar *et al*. 1984															
5	Dormaar *et al*. 1989				-0.0453<br>Ah/0-15<br>(1)						-1.7582<br>(5)					
6	Dormaar *et al*. 1990a								-0.0015<br>Ah/0-15<br>(1)							-0.2524<br>Ah/0-15<br>(2)
7	Dormaar *et al*. 1990b															
8	Dormaar *et al*. 1992															
9	Dormaar *et al*. 1994				0.1122<br>Ah/0-15<br>(2)								0.1979<br>Ah/0-15<br>(1)			
10	Dormaar *et al*. 1997															0.1609<br>Ah/0-15<br>(6)

11	Dormaar *et al.* 1998															
12	Willms *et al.* 1988															
13	Willms *et al.* 1990										-1.6780<br>(15)		-0.0348<br>Ah/0-15<br>(2)			
													-0.0991<br>15-30<br>(2)			
14	Willms *et al.* 2002	-0.1669<br>(2)	-0.8629<br>(2)		-0.0488<br>Ah/0-15<br>(2)		-0.2000<br>(2)	-0.8577<br>(2)	-0.0894<br>Ah/0-15<br>(1)	-0.7540<br>(2)		-1.0737<br>(1)				
15	Henderson *et al.* 2000	-0.2455<br>(9)	-0.9307<br>(8)	0.0090<br>Ah/0-15<br>(9)	0.1094<br>Ah/0-15<br>(9)											
				-0.0203<br>15-30<br>(9)	-0.0337<br>15-30<br>(9)											
				0.0935<br>30-60<br>(9)	0.0943<br>30-60<br>(9)											
					0.0007<br>60-110<br>(9)											
16	Johnston 1961															
17	Smoliak 1965									-0.4971<br>(3)			-0.1951<br>Ah/0-15<br>(2)			
													-0.0608<br>15-30<br>(2)			
18	Smoliak *et al.* 1972			0.0472<br>Ah/0-15<br>(3)	0.1081<br>Ah/0-15<br>(3)								0.3556<br>Ah/0-15<br>(2)			
													-0.0818<br>15-30<br>(2)			
19	Neath *et al.* 1990															
20	Neath *et al.* 1991			-0.1067<br>Ah/0-15<br>(8)								-0.5095<br>(10)	-0.2093<br>Ah/0-15<br>(9)			

21	Chanasyk *et al.* 1995															
22	Lodge 1954				-0.0191<br>Ah/0-15<br>(3)					-0.4126<br>(3)						
23	Bork *et al.* 2012									0.1217<br>(4)		-0.5149<br>(4)				
24	Manley *et al.* 1995															
25	Schuman *et al.* 1999	-0.2978<br>(2)	-0.4746<br>(2)	-0.1968<br>Ah/0-15<br>(2)			-0.2939<br>(2)	-0.5803<br>(5)					-0.2568<br>Ah/0-15<br>(2)			
				0.1666<br>15-30<br>(2)									0.0960<br>15-30<br>(2)			
				0.0506<br>30-60<br>(2)												
26	Ingram *et al.* 2008				0.0550<br>Ah/0-15<br>(2)	-0.1448<br>Ah/0-15<br>(4)			0.0742<br>Ah/0-15<br>(4)	-0.0635<br>(2)		-0.9220<br>(2)				-0.1774<br>Ah/0-15<br>(4)
					-0.0410<br>15-30<br>(2)											
					-0.0137<br>30-60<br>(2)											
27	Shrestha *et al.* 2008				0.0004<br>Ah/0-15<br>(4)	-0.1428<br>Ah/0-15<br>(4)										
28	Frank *et al.* 1995															
29	Wienhold *et al.* 2001				0.2401<br>Ah/0-15<br>(2)				0.2515<br>Ah/0-15<br>(4)							0.4703<br>Ah/0-15<br>(4)
30	Rogers *et al.* 2005				-0.2208<br>Ah/0-15<br>(2)					-0.4525<br>(2)			-0.2828<br>Ah/0-15<br>(2)			

					-0.0377<br>15-30<br>(2)											
31	Haferkamp *et al.* 2004										-1.3636<br>(3)					
32	Haferkamp *et al.* 2005															
33	MacNeil *et al.* 2008									-0.2148<br>(1)		-0.4977<br>(1)				
34	Biondidi *et al.* 1996						0.3088<br>(3)		0.0822<br>Ah/0-15<br>(2)	0.0066<br>(3)				0.0930<br>(6)	-0.1930<br>(6)	0.3556<br>Ah/0-15<br>(5)
35	Biondidi *et al.* 1998				-0.0453<br>Ah/0-15<br>(2)		0.0449<br>(2)			-0.1479<br>(2)				0.1122<br>(2)	0.3109<br>(2)	-0.2483<br>Ah/0-15<br>(1)
36	Shariff *et al.* 1994													0.3803<br>(3)	0.7131<br>(4)	
37	Brand *et al.* 1986									-0.0900<br>(4)			0.1741<br>Ah/0-15<br>(4)			
													0.2270<br>15-30<br>(4)			
38	LeCain *et al.* 2000															
39	Hewins *et al.* 2015									-0.0044<br>(3)		-0.9038<br>(3)				
40	Yang *et al.* 2012									-0.1277<br>(5)		-0.4051<br>(5)				
41	Patton *et al.* 2007									-0.0122<br>(8)						
42	Mousel *et al.* 2011									-0.0868<br>(12)		-0.6530<br>(12)				
43	Reinhart *et al.* 2015									0.0323<br>(2)						
44	Bai *et al.* 2001									-0.1908<br>(8)		-0.5203<br>(7)				

45	Stohlgren *et al*. 1999															
46	Welker *et al*. 2004															

Continuous of Table S2, response ratios for parameters and environmental variables (continued)

		Parameter											Environmental variable			
**No**	**Ref.**	**Shoot C conc.**	**Shoot N conc.**	**Root C conc.**	**Litter C conc.**	**Liter N conc.**	**Litter C/N ratio**	**Soil C conc.**	**Soil N conc.**	**Soil C/N ratio**	**Soil NH4$^+$ conc.**	**Soil NO3$^-$ conc.**	**BD**	**Soil Moisture**	**Soil Temp.**	**Soil PH**
1	Li *et al.* 2009		0.1696<br>(2)			-0.1600<br>(2)										
2	Li *et al.* 2012							-0.2634<br>Ah/0-15<br>(2)			0.2965<br>Ah/0-15<br>(2)	0.4848<br>Ah/0-15<br>(2)	0.2188<br>Ah/0-15<br>(3)			
								-0.1551<br>15-30<br>(2)					0.0512<br>15-30<br>(2)			
								-0.0540<br>30-60<br>(4)					-0.0163<br>30-60<br>(4)			
3	Dormaar *et al*. 1977								0.1042<br>Ah/0-15<br>(2)	0.2259<br>Ah/0-15<br>(1)	-0.0163<br>Ah/0-15<br>(1)					0.1287<br>Ah/0-15<br>(2)
4	Dormaar *et al.* 1984							0.1433<br>Ah/0-15<br>(6)								
5	Dormaar *et al.* 1989								-0.0920<br>Ah/0-15<br>(1)	-0.0241<br>Ah/0-15<br>(1)		-0.1193<br>Ah/0-15<br>(1)	0.1331<br>Ah/0-15<br>(4)	-0.2151<br>Ah/0-15<br>(1)		
6	Dormaar *et al.* 1990a							0.0000<br>Ah/0-15<br>(1)								
7	Dormaar *et al.* 1990b										0.4983<br>Ah/0-15<br>(1)		0.1014<br>Ah/0-15<br>(1)		0.1800<br>Ah/0-15<br>(3)	
8	Dormaar *et al.* 1992				0.0101<br>(2)	0.3289<br>(1)	-0.5492<br>(12)		0.0725<br>Ah/0-15<br>(2)							0.0077<br>Ah/0-15<br>(2)

9	Dormaar *et al.* 1994							0.1122<br>Ah/0-15<br>(2)	0.1305<br>Ah/0-15<br>(2)	-0.0180<br>Ah/0-15<br>(2)	-0.2697<br>Ah/0-15<br>(1)	0.1709<br>Ah/0-15<br>(2)	0.0179<br>Ah/0-15<br>(4)	-0.6420<br>Ah/0-15<br>(1)		0.0193<br>Ah/0-15<br>(2)
10	Dormaar *et al.* 1997															
11	Dormaar *et al.* 1998								0.1188<br>Ah/0-15<br>(3)		0.5330<br>Ah/0-15<br>(3)	0.6546<br>Ah/0-15<br>(5)	0.1770<br>Ah/0-15<br>(8)	-0.5393<br>Ah/0-15<br>(1)		0.0799<br>Ah/0-15<br>(3)
12	Willms *et al.* 1988										0.1810<br>Ah/0-15<br>(1)	0.1696<br>Ah/0-15<br>(3)				
13	Willms *et al.* 1990							-0.1488<br>Ah/0-15<br>(1)	-0.1398<br>Ah/0-15<br>(1)		-0.4249<br>Ah/0-15<br>(1)	0.1074<br>Ah/0-15<br>(2)				
14	Willms *et al.* 2002	-0.0044<br>(3)	-0.0201<br>(3)		-0.0068<br>(3)	0.0161<br>(3)	-0.0209<br>(3)	-0.0131<br>Ah/0-15<br>(2)	-0.0258<br>Ah/0-15<br>(2)	0.0128<br>Ah/0-15<br>(2)			0.0432<br>Ah/0-15<br>(2)	-0.4038<br>Ah/0-15<br>(2)		0.0069<br>Ah/0-15<br>(2)
15	Henderson 2000							0.0280<br>Ah/0-15<br>(7)		-0.0057<br>Ah/0-15<br>(9)			0.1060<br>Ah/0-15<br>(9)			
								-0.0234<br>15-30<br>(7)					0.0303<br>15-30<br>(9)			
								0.1627<br>30-60<br>(7)					-0.0252<br>30-60<br>(9)			
								-0.0126<br>60-110<br>(8)								
16	Johnston 1961														0.0527<br>Ah/0-15<br>(3)	
17	Smoliak 1965															
18	Smoliak *et al.* 1972			-0.0277<br>Ah/0-15<br>(1)				0.0488<br>Ah/0-15<br>(2)				0.4235<br>Ah/0-15<br>(3)	-0.0001<br>Ah/0-15<br>(3)	-0.3345<br>Ah/0-15<br>(3)		-0.0401<br>Ah/0-15<br>(2)

19	Neath *et al.* 1990b												0.0041<br>Ah/0-15<br>(12)			
													-0.0090<br>15-30<br>(12)			
													-0.0073<br>30-60<br>(29)			
20	Neath *et al.* 1991			0.0945<br>Ah/0-15<br>(10)	-0.0017<br>(7)			0.2124<br>Ah/0-15<br>(20)								
21	Chanasyk *et al.* 1995													0.2222<br>Ah/0-15<br>(6)		
22	Lodge 1954							-0.0191<br>Ah/0-15<br>(3)					0.0700<br>Ah/0-15<br>(4)	-0.2610<br>Ah/0-15<br>(4)		0.0273<br>Ah/0-15<br>(4)
23	Bork *et al*. 2012							0.0940<br>Ah/0-15<br>(3)								
24	Manley *et al.* 1995							0.1076<br>Ah/0-15<br>(4)					0.0761<br>Ah/0-15<br>(4)			
													-0.0211<br>15-30<br>(2)			
													-0.0211<br>30-60<br>(4)			
25	Schuman *et al*. 1999	-0.0114<br>(2)	-0.0040<br>(2)	0.0586<br>Ah/0-15<br>(2)	0.1168<br>(2)	0.0383<br>(2)	0.0777<br>(2)									
26	Ingram *et al*. 2008							0.0602<br>Ah/0-15<br>(4)	0.0422<br>Ah/0-15<br>(2)	0.0000<br>Ah/0-15<br>(2)						

								0.0448<br>15-30<br>(2)								
								-0.0516<br>30-60<br>(2)								
27	Shrestha *et al*. 2008							-0.0381<br>Ah/0-15<br>(4)		-0.0127<br>Ah/0-15<br>(4)						
28	Frank *et al*. 1995							-0.0457<br>Ah/0-15<br>(4)		0.2321<br>Ah/0-15<br>(1)						
								-0.1827<br>15-30<br>(4)								
								-0.1189<br>30-60<br>(2)								
								-0.1119<br>60-110<br>(2)								
29	Wienhold *et al*. 2001							-0.0274<br>Ah/0-15<br>(4)	-0.0172<br>Ah/0-15<br>(2)	-0.0649<br>Ah/0-15<br>(2)	0.4864<br>Ah/0-15<br>(4)	0.2236<br>Ah/0-15<br>(2)		0.2514<br>Ah/0-15<br>(2)		-0.0083<br>Ah/0-15<br>(4)
30	Rogers *et al*. 2005															
31	Haferkamp *et al*. 2004															
32	Haferkamp *et al*. 2005		0.1372<br>(2)													
33	MacNeil *et al*. 2008															
34	Biondidi *et al*. 1996		0.3183<br>(3)													

35	Biondidi *et al.* 1998		0.1928 (2)													
36	Shariff *et al.* 1994															
37	Brand *et al.* 1986															
38	LeCain *et al.* 2000															
39	Hewins *et al.* 2015							-0.1401 Ah/0-15 (3)	-0.0617 Ah/0-15 (3)					-0.1282 Ah/0-15 (6)		
								-0.0852 15-30 (3)								
40	Yang *et al.* 2012															
41	Patton *et al.* 2007															
42	Mousel *et al.* 2011															
43	Reinhart *et al*. 2015															
44	Bai *et al*. 2001															
45	Stohlgren *et al*. 1999							-0.3103 Ah/0-15 (4)								
46	Welker *et al*. 2004							0.3383 Ah/0-15 (2)	0.3710 Ah/0-15 (2)					-0.0546 Ah/0-15 (1)	0.1448 Ah/0-15 (1)	
								0.5306 15-30 (1)						0.1919 15-30 (2)		

Fig. S1. The frequency distributions of the natural logarithm of response ratios (ln*RR*) for pool variables. The solid curve is a Gaussian distribution fitted to the frequency data. The x axis is ln*RR* and y axis is frequency. The vertical dash line is at ln*RR* = 0. *P* is the probability that the mean of ln*RR* equals zero. ln*RR* of root C stock (30-60 cm), soil C stock (60-110 cm) and soil microbial biomass carbon (SMBC) were not fit Gaussian function therefore not report here.

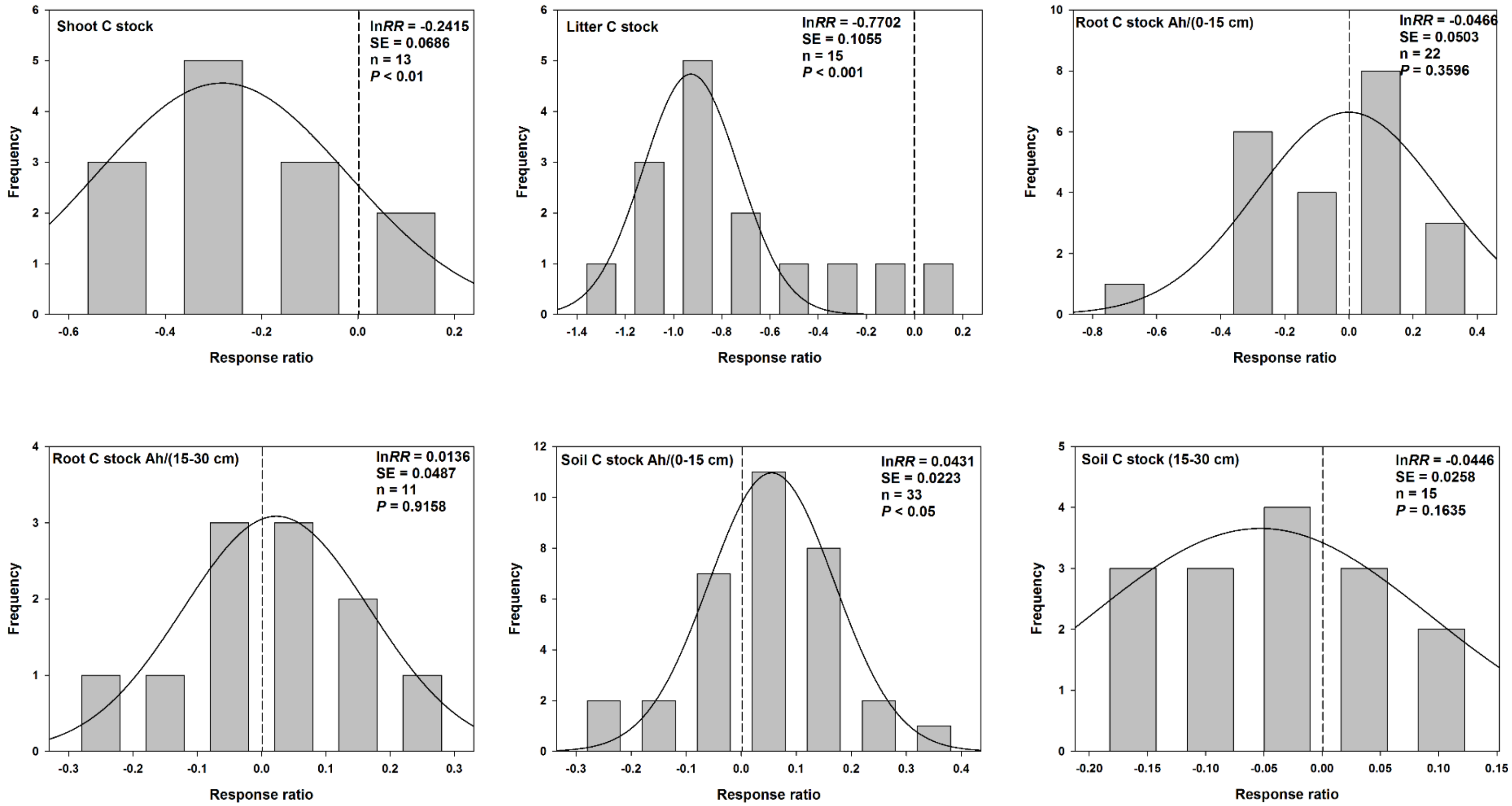

Fig. S1. To be continued

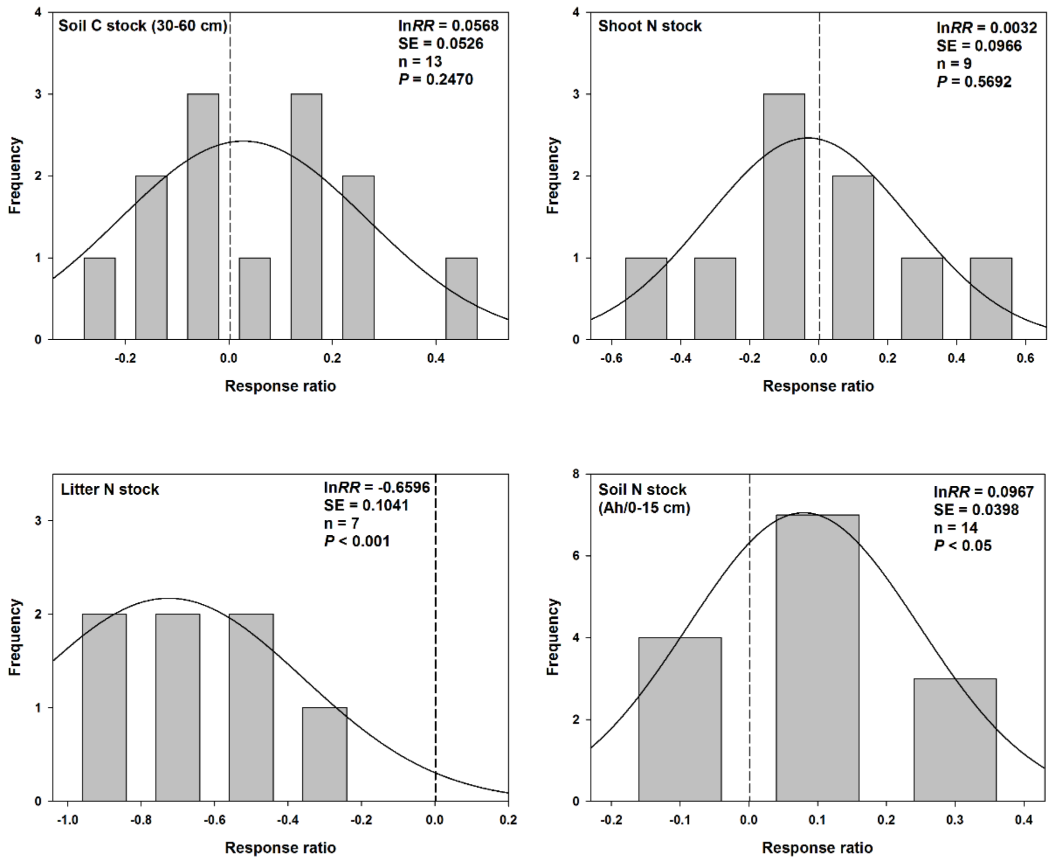
Soil C stock (30-60 cm)
ln*RR* = 0.0568
SE = 0.0526
n = 13
P = 0.2470
Frequency
Response ratio
Shoot N stock
ln*RR* = 0.0032
SE = 0.0966
n = 9
P = 0.5692
Litter N stock
ln*RR* = -0.6596
SE = 0.1041
n = 7
P < 0.001
Soil N stock
(Ah/0-15 cm)
ln*RR* = 0.0967
SE = 0.0398
n = 14
P < 0.05

Fig. S2. The frequency distributions of the natural logarithm of response ratios (ln*RR*) for fluxes variables. The solid curve is a Gaussian distribution fitted to the frequency data. The x axis is ln*RR* and y axis is frequency. The vertical dash line is at ln *RR* = 0. *P* is the probability that the mean of ln *RR* equals zero. ln*RR* of root biomass (15-30 cm), litter decomposition % and root decomposition % were not fit Gaussian function therefore not report here.

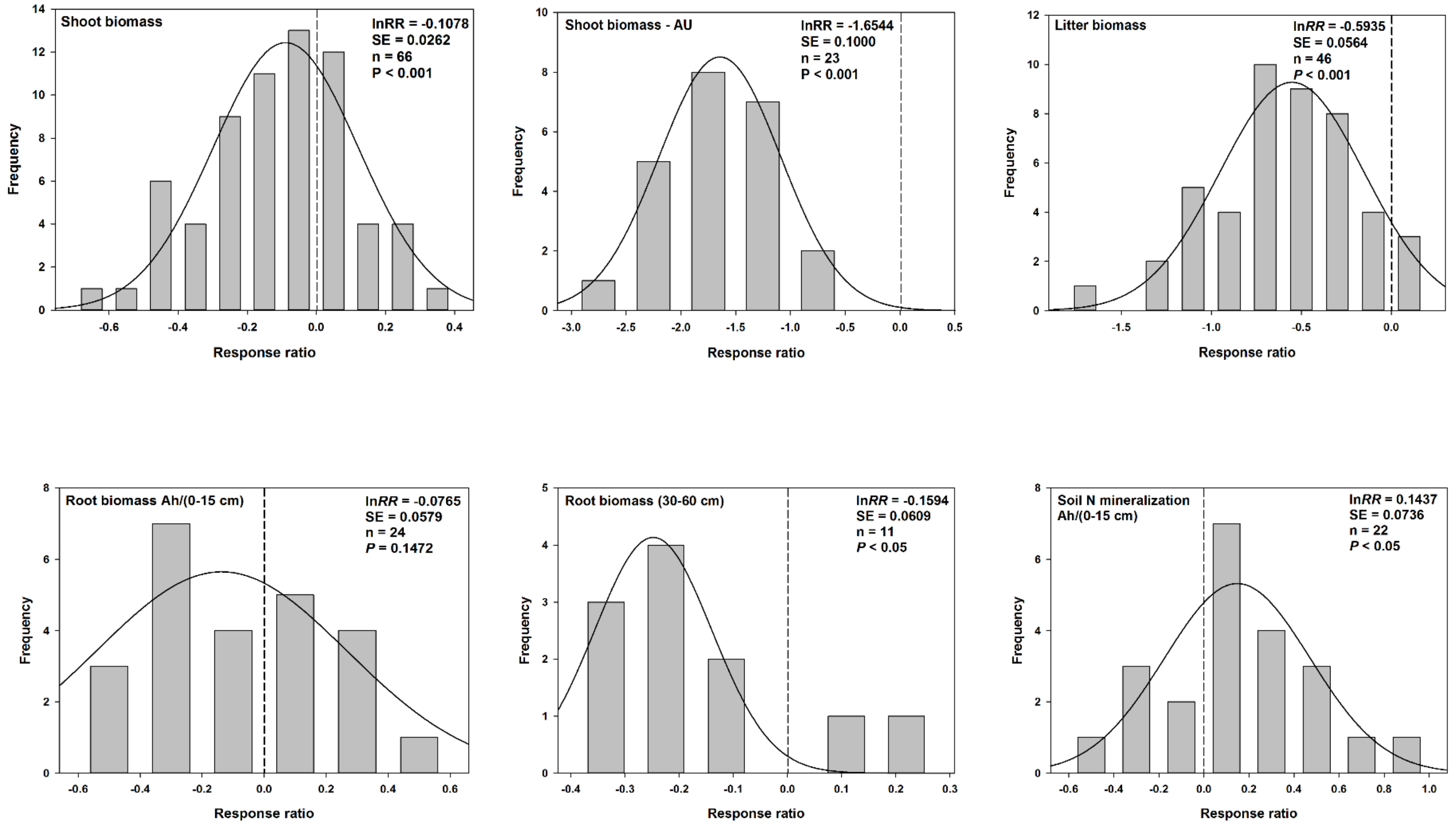

Fig. S3. The frequency distributions of the natural logarithm of response ratios (ln*RR*) for parameter variables. The solid curve is a Gaussian distribution fitted to the frequency data. The x axis is ln *RR* and y axis is frequency. The vertical dash line is at ln *RR* = 0. *P* is the probability that the mean of ln *RR* equals zero. ln*RR* of shoot C concentration and soil C concentration (30-60 cm) were not fit Gaussian function therefore not report here.

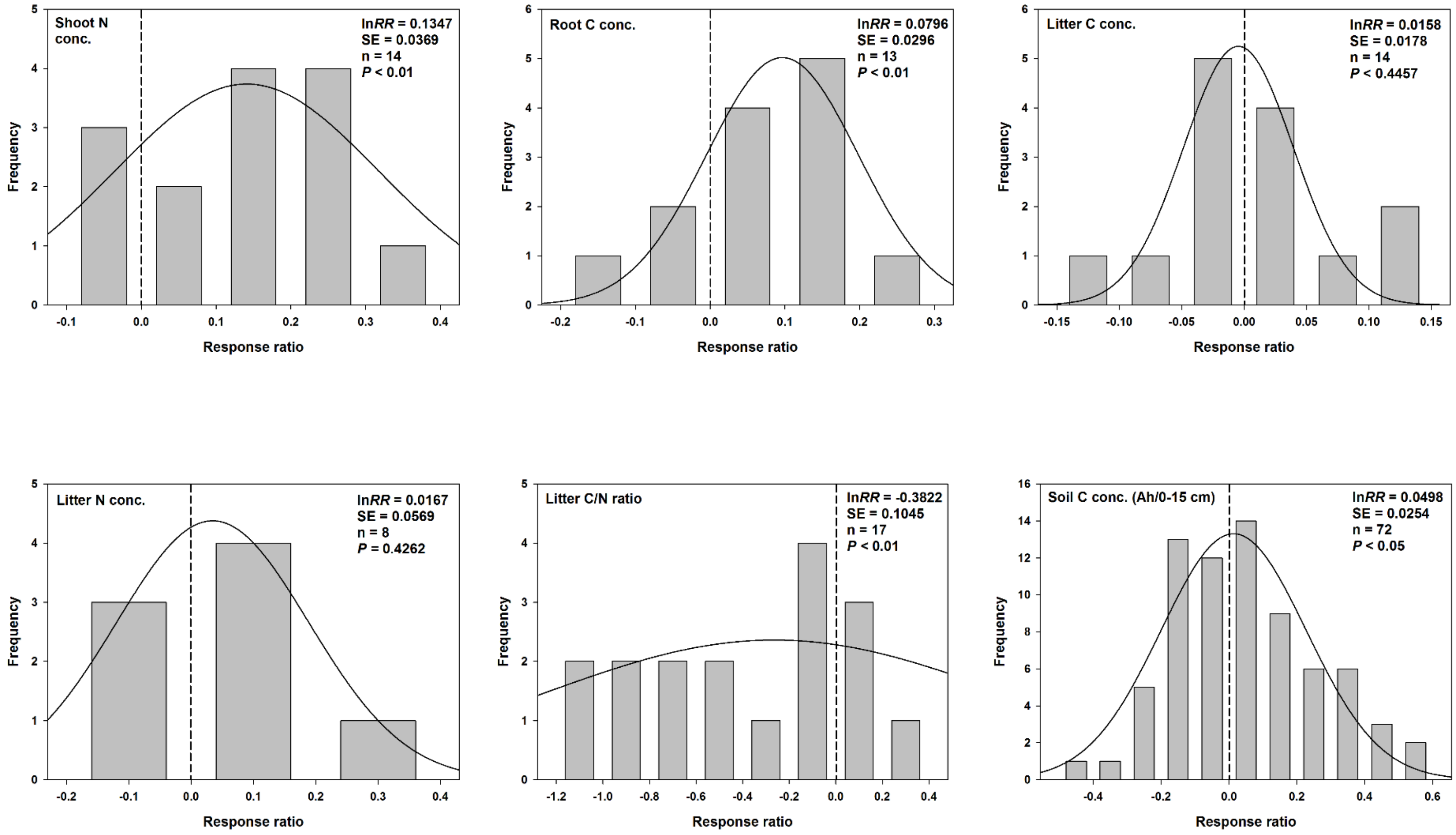

Fig. S3. To be continued

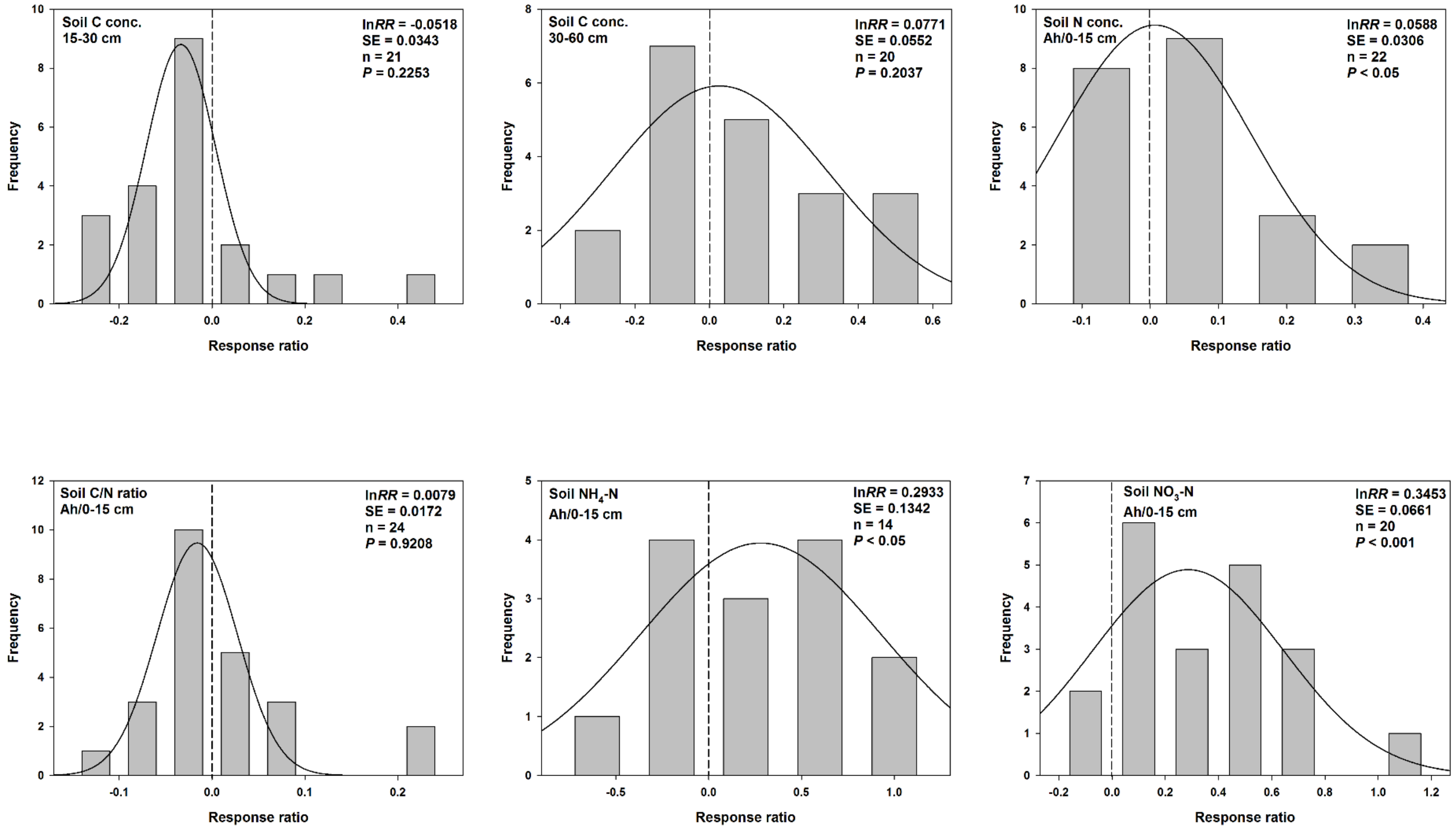

Soil C conc.
15-30 cm
lnRR = -0.0518
SE = 0.0343
n = 21
P = 0.2253
Frequency
Response ratio
Soil C conc.
30-60 cm
lnRR = 0.0771
SE = 0.0552
n = 20
P = 0.2037
Frequency
Response ratio
Soil N conc.
Ah/0-15 cm
lnRR = 0.0588
SE = 0.0306
n = 22
P < 0.05
Frequency
Response ratio
Soil C/N ratio
Ah/0-15 cm
lnRR = 0.0079
SE = 0.0172
n = 24
P = 0.9208
Frequency
Response ratio
Soil NH4-N
Ah/0-15 cm
lnRR = 0.2933
SE = 0.1342
n = 14
P < 0.05
Frequency
Response ratio
Soil NO3-N
Ah/0-15 cm
lnRR = 0.3453
SE = 0.0661
n = 20
P < 0.001
Frequency
Response ratio

Fig. S4. The frequency distributions of the natural logarithm of response ratios (ln*RR*) for environmental variables. The solid curve is a Gaussian distribution fitted to the frequency data. The x axis is ln *RR* and y axis is frequency. The vertical dash line is at ln *RR* = 0. *P* is the probability that the mean of ln *RR* equals zero.

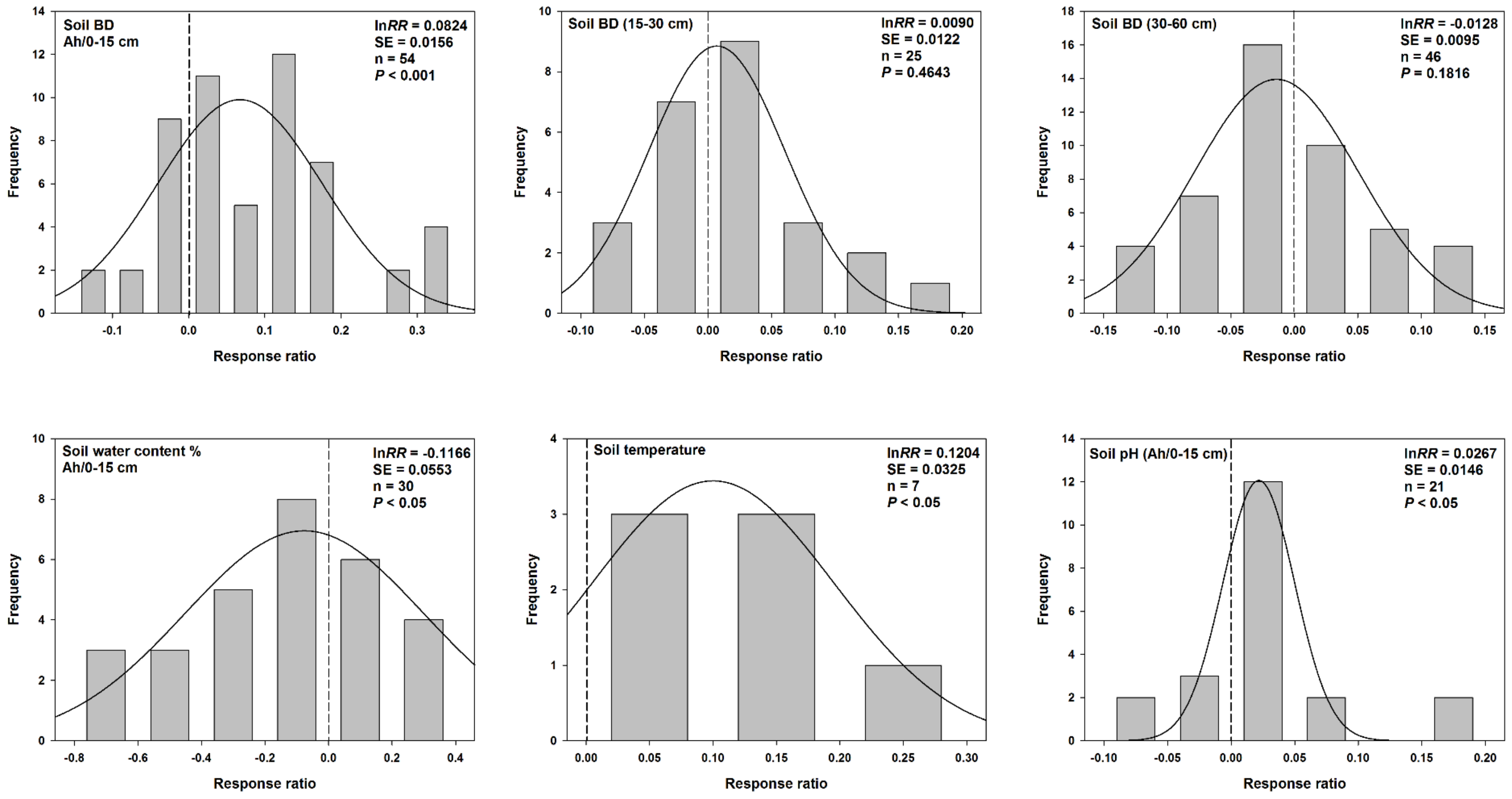

Supplement: Supplementary Information [file srep33190-s1.pdf]
